# Near-Comparable Frequency of rob(21;21) and rob(14;21) Translocations in Trisomy 21: A Retrospective Cytogenetic Analysis of 9,313 Cases From an Indian Referral Cohort

**DOI:** 10.7759/cureus.104606

**Published:** 2026-03-03

**Authors:** Suryaprakash Kunda, Mukkanteswararao Kasaragadda, Sambasivarao Patibandla, Shalini Singh, Sidrah Parvez, Tirupathi Rao Golla

**Affiliations:** 1 Genetics, American Institute of Pathology and Laboratory Sciences Private Limited (AMPATH) Labs, Hyderabad, IND; 2 Biotechnology, Era's Lucknow Medical College and Hospital, Lucknow, IND; 3 Conservation Genetics Lab, Council of Scientific and Industrial Research (CSIR) - Centre for Cellular and Molecular Biology, Hyderabad, IND

**Keywords:** abnormal karyotypes, chromosomal abnormality, down syndrome, gender, reproductive failure

## Abstract

Background

Chromosomal abnormalities are one of the leading causes of developmental delay, congenital anomalies, and reproductive failure. Cytogenetic evaluation aids diagnosis, genetic counselling, and prevention. This study evaluated the cytogenetic and demographic profile of chromosomal abnormalities, with a focus on Down syndrome (DS, trisomy 21), in a large referral cohort.

Methodology

A retrospective analysis of 9,313 blood samples was performed using GTG-banding karyotyping. Clinical indications, age, sex, and diagnostic yield were evaluated to identify frequency patterns and etiological trends.

Results

Among 9,313 samples analyzed, 1,597 (17.1%) were diagnosed with DS. A male predominance was observed, with 968 (60.6%) males and 629 (39.4%) females. Classical free trisomy 21 (47,XX,+21/47,XY,+21) comprised 1,465 (91.7%) cases, while Robertsonian translocations accounted for 90 (5.6%). Other forms, including mosaicism and double aneuploidy, constituted 42 (2.6%). Among Robertsonian cases, rob(14;21) and rob(21;21) were the most frequent subtypes. In most cases, 933 (58%) were diagnosed within the first six months of life. Referral indications included clinical suspicion of trisomy 21 in 605 (38%) cases, unassigned clinical history in 460 (29%) cases, prenatal screening in 220 (14%) cases, anatomical anomalies in 75 (5%) cases, abnormal antenatal ultrasound findings in 86 (5%) cases, disorders of sex development in 16 (1%) cases, and other indications in 135 (8%) cases.

Conclusions

DS remains the most common autosomal aneuploidy, primarily resulting from meiotic nondisjunction. The observed male predominance and increasing referrals based on prenatal screening highlight the continued importance of cytogenetic evaluation in clinical practice. Integration of cytogenetic findings with clinical assessment is essential for accurate diagnosis, genetic counselling, and informed reproductive decision-making. The identification of Robertsonian translocations in this cohort reinforces the need for parental karyotyping to determine whether the rearrangement is inherited or de novo. In our practice, parental karyotyping is recommended in all cases of trisomy 21 to distinguish classical (free) trisomy from translocation-associated trisomy 21, thereby enabling accurate recurrence risk assessment in future pregnancies.

## Introduction

Trisomy 21, which is commonly known as Down syndrome (DS), is the most common chromosomal/genetic disorder, characterized by the presence of an extra copy of chromosome 21 [1]. This extra chromosome 21 leads to this syndrome by creating gene dosage imbalances in the 21q22 region, which in turn interferes with normal developmental processes. This most common viable autosomal trisomy is the leading genetic cause for intellectual disability worldwide, with an incidence rate ranging from 1 in 850 to 1 in 1,000 live births [2].

Although DS affects all ethnic and racial groups, its prevalence varies across the globe due to different distributions of maternal age. The prevalence of DS is strongly associated with the age of the mother at the time of conception. Published data clearly indicate that DS is associated with various other maternal risk factors, in addition to maternal age. Methionine synthase reductase (MTRR) G- polymorphism has been found to be one such maternal risk factor associated with DS births [3]. Other non-genetic factors include consanguineous marriages, drug exposure, environmental toxins, and reproductive health, which increase the risk of DS [4].

It is estimated that nearly one in 365 fetuses carries this condition at 10 weeks of gestation [5]. The cytogenetic mechanisms underlying DS include free trisomy 21, duplication of the DS critical region, Robertsonian translocations, and other structural rearrangements involving chromosome 21 [6]. Among these, free (complete) trisomy 21 accounts for approximately 95% of cases and results from meiotic nondisjunction, with nearly 80% of cases arising during maternal meiosis and being associated with maternal age greater than 35 years [2,7]. The extra chromosome 21 has been shown to originate predominantly from maternal meiotic nondisjunction events [8]. Robertsonian translocations occur in approximately 3-4% of cases and involve the attachment of chromosome 21 to another acrocentric chromosome, most commonly chromosome 14, although chromosomes 13, 15, 21, and 22 may also be involved. These translocations may occur de novo or be inherited from a parent carrying a balanced translocation, a distinction that is clinically important because inherited forms confer a high recurrence risk in subsequent pregnancies [8]. Recurrence risk is estimated to be 10-15% when the carrier is the mother and 1%-3% when the carrier is the father. Therefore, parental karyotyping following a diagnosis of DS is essential for accurate recurrence risk assessment and appropriate genetic counseling. Mosaic trisomy 21 represents approximately 1%-2% of cases and arises from nondisjunction during early embryonic mitosis rather than meiosis [9].

However, there is currently no definitive treatment for DS; researchers are focusing on combining clinical and translational approaches to better understand the molecular pathways involved in DS and to manage associated health conditions, to enhance both longevity and quality of life. While preliminary diagnosis may be based on clinical features or prenatal screening, definitive diagnosis of DS relies on cytogenetic evaluation, which remains the gold standard for visualizing chromosomal abnormalities [1,10,11]. Rapid molecular techniques such as fluorescence in situ hybridization (FISH) and quantitative fluorescent PCR (QF-PCR) are also used for prenatal detection of common chromosomal aneuploidies; however, conventional karyotyping remains essential for identifying balanced structural rearrangements [10]. GTG-banding has been one of the cornerstone techniques for cytogenetic diagnosis for several years and allows identification of chromosomal number and structural abnormalities through distinct banding patterns visualized by microscopy.

In India, despite the high detection rate by various antenatal screening programs, it is still the most common genetic disorder, with an incidence rate of 0.88 to 1.09 per 1,000 live births [12]. Previous studies from India have also reported regional variability in the cytogenetic profile of DS across different populations, including tribal cohorts [13]. We conducted this retrospective study to evaluate the present status of DS in South Central India and understand its profile, frequency, and pattern in this region. This was a very large study wherein 9,313 samples were evaluated during a time span of eight years.

## Materials and methods

Materials and methods

Study Design and Samples

This retrospective study included data from 9,313 unique patients who were clinically suspected of having DS, along with other clinical indications, and were referred to the American Institute of Pathology and Laboratory Sciences Private Limited (AMPATH), Hyderabad, over eight years from January 2017 to October 2025 for cytogenetic evaluation. The inclusion criteria comprised all consecutive patients referred for peripheral blood karyotyping during the study period.

Peripheral venous blood (2 mL) was collected in heparinized vacutainers, and only one sample per patient was included in the final analysis. No prenatal invasive samples (amniotic fluid or chorionic villus samples) were included in this study; all analyses were performed on postnatal peripheral blood specimens. In routine laboratory practice, if initial cultures demonstrated inadequate growth or insufficient metaphase yield, a repeat sample was requested to ensure reliable karyotype interpretation. Therefore, the final cohort of 9,313 represents successfully analyzed cases, and no samples were excluded due to culture failure in the analyzed dataset.

Cytogenetic Analysis

Chromosome analysis was performed in cases clinically suspected of DS using the peripheral blood lymphocyte culture technique described by Moorhead et al. [14]. Briefly, 0.5 mL of heparinized peripheral blood was used for lymphocyte culture in RPMI-1640 medium supplemented with phytohemagglutinin and incubated at 37 °C for 72 hours. Metaphase arrest was induced using 1% colchicine, followed by hypotonic treatment with 0.075 M KCl and fixation in methanol:acetic acid (3:1), as described by Hungerford [15]. Slides were prepared and stained using the Giemsa-Trypsin-Giemsa (GTG) banding method as described by Seabright [16].

Chromosome analysis was performed in cases clinically suspected of DS using the peripheral blood lymphocyte culture technique described by Moorhead et al. [14]. From the collected 2 mL peripheral blood sample, two parallel lymphocyte cultures were set up, each using 0.5 mL of heparinized blood in RPMI-1640 medium supplemented with phytohemagglutinin and incubated at 37 °C for 72 hours. Metaphase arrest was induced using 1% colchicine, followed by hypotonic treatment with 0.075 M KCl and fixation in methanol:acetic acid (3:1), as described by Hungerford [15]. Slides were prepared and stained using the GTG banding technique as described by Seabright [16]. A minimum of 20 metaphases were analyzed per case. In cases where mosaicism was suspected, at least 50 metaphases were evaluated to confirm the diagnosis. Karyotypes were reported following standard cytogenetic criteria.

The culture setup was performed by two trained technicians, and slides were prepared independently to minimize technical variability and maintain quality control. Chromosome analysis was carried out using a bright-field microscope with the assistance of the IKAROS imaging system (Carl Zeiss, Oberkochen, Germany). Karyotype interpretation was performed in accordance with the International System for Human Cytogenomic Nomenclature (ISCN) guidelines in effect at the time of analysis (ISCN 2016, 2020, and 2024 editions, as applicable). Clinical and laboratory data were retrieved retrospectively from medical records. Data were analyzed using SPSS version 23 (IBM Corp., Armonk, NY), and descriptive statistics, including mean, standard deviation, frequency, and percentage, were used to summarize the variables.

Ethical Considerations

This retrospective study involved the analysis of anonymized cytogenetic data obtained during routine clinical care. No direct patient interaction or study-related intervention was undertaken. In accordance with institutional policy and prevailing ICMR guidelines, formal ethical committee approval and individual informed consent were waived by the internal review committee of AMPATH Labs (IRC/AMPATH/02/2026) due to the retrospective and anonymized nature of the study. Patient confidentiality and data privacy were strictly maintained throughout. The study was conducted in accordance with institutional ethical guidelines and the principles outlined in the Declaration of Helsinki.

## Results

**Figure 1:**
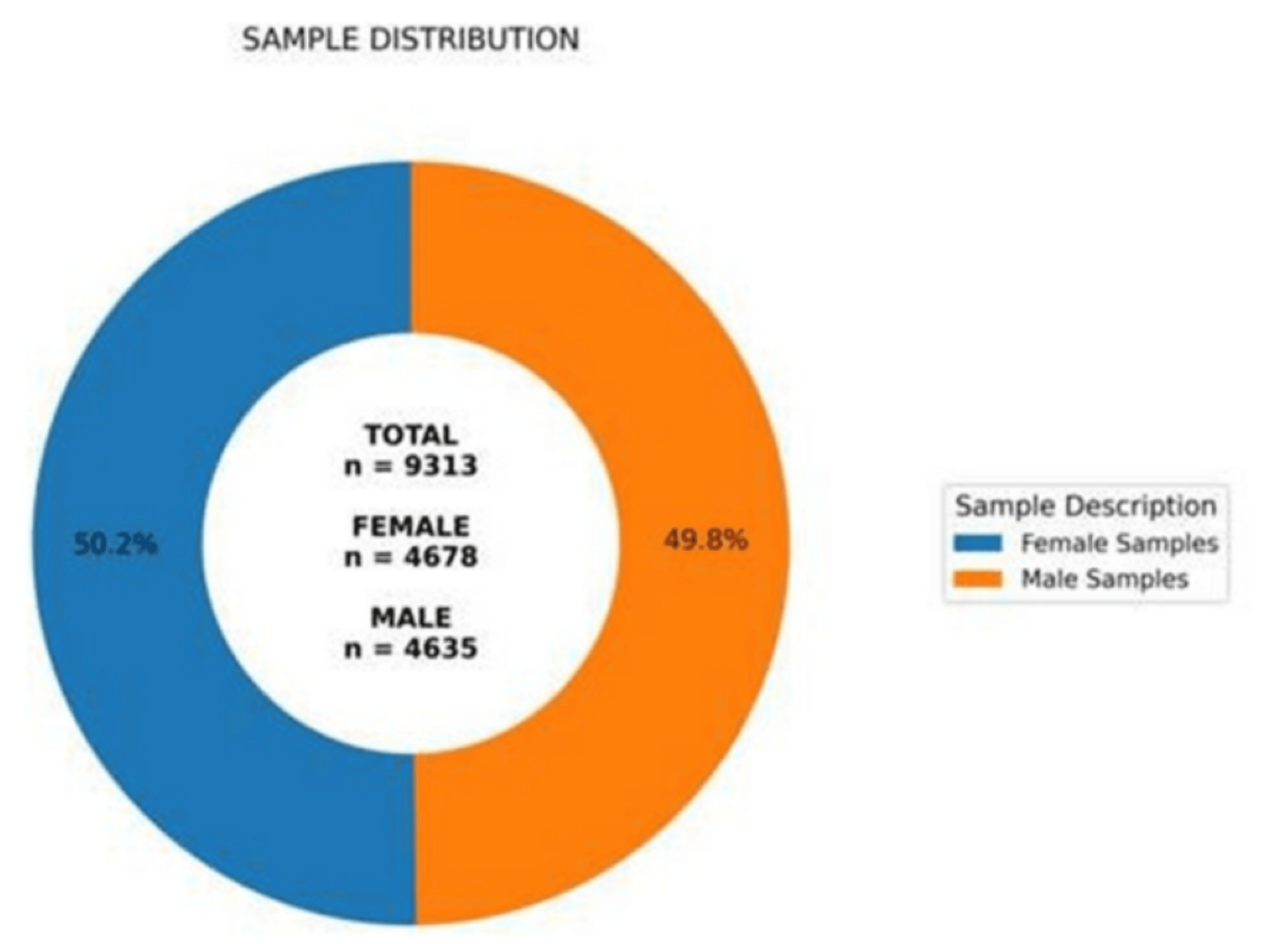
Cytogenetic analysis was performed on 9,313 cases suspected of DS, among which 4,678 (50.2%) were female and 4,635 (49.8%) were male.

A total of 9,313 samples clinically suspected of DS were analyzed cytogenetically, of which 4,678 (50.2%) were female, and 4,635 (49.8%) were male (Figure 1).

Cytogenetic analysis revealed that 1,597 cases (17.1%) were diagnosed with DS (trisomy 21). The age of patients diagnosed with DS ranged from birth to over 20 years. The majority of cases (*n* = 933, 58.0%) were diagnosed within the first six months of life, followed by 268 (16.78%) between six months and one year, and 312 (19.54%) between one and five years. Smaller proportions were diagnosed between five and 10 years (*n* = 54, 3.38%), 10 and 20 years (*n* = 27, 1.69%), and above 20 years of age (*n* = 3, 0.19%). The overall sex distribution among the 1,597 confirmed DS patients showed that 968 (60.6%) were male and 629 (39.4%) were female.

**Figure 2:**
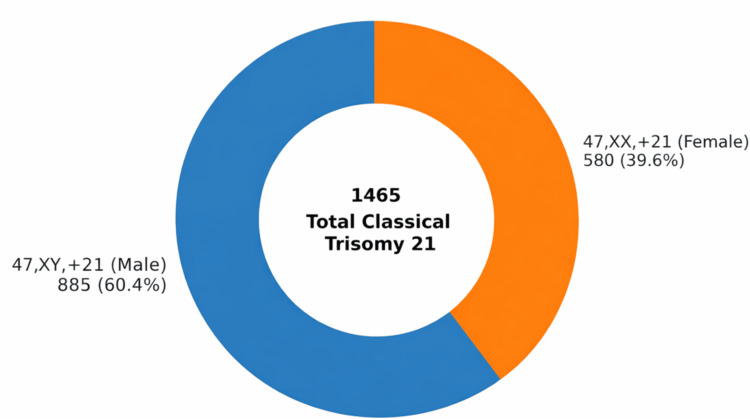
Sex-wise distribution of classical (free) trisomy 21 karyotypes (47,XY,+21 and 47,XX,+21) identified in the study cohort. Among the 1,465 classical trisomy 21 cases analyzed, 885 (60.4%) were males and 580 (39.6%) were females. Values are presented as *n* (%). The distribution demonstrates a male predominance in classical trisomy 21 cases. Blue indicates male classical trisomy 21 cases (47,XY,+21), and orange indicates female classical trisomy 21 cases (47,XX,+21).

Classical free trisomy 21 (47,XX,+21 or 47,XY,+21) accounted for 1,465 (91.7%) of all confirmed DS cases. The sex-wise distribution of classical trisomy 21 cases is illustrated in Figure 2.

Classical free trisomy 21 (47,XX,+21 or 47,XY,+21) accounted for 1,465 (91.7%) of all confirmed DS cases. The sex-wise distribution of classical trisomy 21 cases is illustrated in Figure 2. A rare double aneuploidy, 48,XXY,+21, was detected in 2 (0.13%) cases.

**Figure 3:**
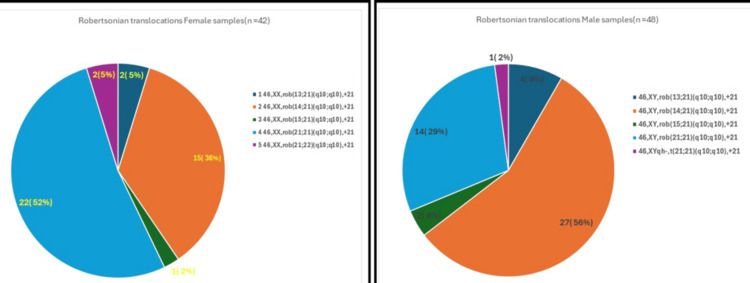
Sex-wise distribution of Robertsonian translocation subtypes in trisomy 21 cases (male: n = 48; female: n = 42). A total of 90 Robertsonian translocation cases were analyzed, including 48 (53.3%) males and 42 (46.7%) females. Distribution of Robertsonian translocation subtypes in trisomy 21 cases by sex. The left pie chart represents female samples (*n* = 42), and the right pie chart represents male samples (*n* = 48). Categories include rob(13;21), rob(14;21), rob(15;21), rob(21;21), and rob(21;22). Numbers within the segments indicate case counts with corresponding percentages (*n*, %)

Robertsonian translocations were identified in 90 cases (5.6%) of the cohort. Among these, rob(14;21)(q10;q10),+21 was the most frequent subtype, observed in 42 (2.63%) cases, followed by rob(21;21)(q10;q10),+21 in 36 (2.25%) cases. Less frequent Robertsonian subtypes included rob(13;21)(q10;q10),+21 in 6 (0.4%) cases, rob(15;21)(q10;q10),+21 in 3 (0.2%) cases, rob(21;22)(q10;q10),+21 in 2 (0.1%) cases, and one case (0.1%) of t(21;21) associated with heterochromatic polymorphism.The distribution of Robertsonian translocation subtypes stratified by sex is illustrated in Figure 3.

Pericentric inversions, including inv(9)(p11;q13) and inv(Y)(p11;q13), were observed in 10 (0.63%) and 8 (0.50%) cases, respectively.

Among female patients, the most frequent finding was classical free trisomy 21 (47,XX,+21), accounting for 580 cases.

Robertsonian translocations were identified in 42 (2.6%) female cases and included 46,XX,rob(14;21)(q10;q10),+21 in 15 (0.9%) cases and 46,XX,rob(21;21)(q10;q10),+21 in 22 (1.4%) cases. Less frequent Robertsonian subtypes comprised 46,XX,rob(13;21)(q10;q10),+21 in 2 (0.1%) cases, 46,XX,rob(15;21)(q10;q10),+21 in 1 (0.1%) case, and 46,XX,rob(21;22)(q10;q10),+21 in 2 (0.1%) cases.

Other trisomy 21 karyotypes in females included chromosomal polymorphic findings such as 47,XX,+21,15pstk+ observed in 1 (0.1%) case and 47,XX,9qh+,+21 observed in 3 (0.2%) cases.

Among males, the predominant karyotype was classical free trisomy 21 (47,XY,+21), accounting for 885 (55.4%) cases.

Robertsonian translocations were identified in 48 (3.0%) male cases and included 46,XY,rob(14;21)(q10;q10),+21 in 27 (1.7%) cases, 46,XY,rob(21;21)(q10;q10),+21 in 14 (0.9%) cases, 46,XY,rob(13;21)(q10;q10),+21 in 4 (0.3%) cases, 46,XY,rob(15;21)(q10;q10),+21 in 2 (0.1%) cases, and 1 (0.1%) case of t(21;21) associated with heterochromatic polymorphism.

Other trisomy 21 karyotypes in males included pericentric inversions such as 47,XY,inv(9)(p11;q13),+21 observed in 10 (0.6%) cases and additional rare structural rearrangements, including 47,XY,del(5)(p15.3),+21; 47,XY,inv(14)(p13;q12),+21; and 47,XY,inv(6)(p25;q12),+21, each observed in 1 (0.1%) case.

Mosaic forms of DS were also identified. Karyotypes such as mos 47,XY,+21/46,XY and mos 47,XX,+21/46,XX were observed, with a total of nine mosaic cases recorded across both sexes. Additionally, a rare double aneuploidy, 48,XXY,+21, was detected in two individuals, representing cytogenetic coexistence of trisomy 21 and an additional X chromosome.

When examining the distribution across age groups, the highest number of cases (933) was observed in the six-month age category, highlighting that most diagnoses are made early in life. Most Trisomy 21 cases (58%) were diagnosed within the first six months of life, with 268 (16.78%) at six months to one year. This was followed by 312 cases (19.54%) in the one- to five-year group, 54 (3.38%) in the five- to 10-year range, 27 (1.69%) in the 10- to 20-year range, and only three cases (0.19%) in individuals over 20 years (Table 1). Referral indications included unassigned referrals in 460 (29%) cases, prenatal screening in 220 (14%) cases, anatomical defects in 75 (5%) cases, abnormal antenatal ultrasound findings in 86 (5%) cases, disorders of sex development in 16 (1%) cases, and other indications in 135 (8%) cases (Table 2).

**Table 1:** Samples categorized by age. Age-wise distribution of confirmed Down syndrome (Trisomy 21) cases in the study cohort (N = 1,597). Values represent the total number of cases and corresponding percentages across predefined age groups: 0-6 months, 6 months to 1 year, 1-5 years, 5-10 years, 10-20 years, and ≥20 years. The distribution reflects the entire cohort and was not stratified by sex.

S. no.	Age group	Number of cases	Percentage (%)
1	0-6 months	933	58.00%
2	6 months-1 year	268	16.78%
3	1-5 years	312	19.54%
4	5-10 years	54	3.38%
5	10-20 years	27	1.69%
6	≥20 years	3	0.19%
	Total	1,597	100%

**Table 2:** Samples categorized by clinical history. Distribution of referral indications among confirmed Down syndrome (Trisomy 21) cases (N = 1,597). Clinical indication categories include anatomical anomalies, disorders of sex development (DSD), unassigned referrals, other indications, prenatal screening, clinical suspicion of Down syndrome phenotype, and abnormal antenatal ultrasound (USG). Values are presented as the number of cases with corresponding percentages (*n*, %).

S. no.	Clinical history	Number of cases	Percentage (%)
1	Anatomical	75	5%
2	DSD	16	1%
3	Unassigned	460	29%
4	Other	135	8%
5	Prenatal screening	220	14%
6	Clinical suspicion of Down phenotype	605	38%
7	Abnormal antenatal ultrasound (USG)	86	5%
	Total	1,597	100%

## Discussion

This cytogenetic analysis evaluated a large cohort of clinically suspected DS cases referred to our diagnostic center, providing insight into the burden and cytogenetic spectrum of DS in this region of India. A wide range of chromosomal alterations underlying DS was identified. Consistent with global epidemiological patterns reporting free trisomy 21 in approximately 95% of affected individuals, classical trisomy 21 predominated in our cohort (91.7%) [6,7]. Among male participants, the karyotype 47,XY,+21 accounted for 60.6% of cases, while 47,XX,+21 represented 39.4% of female cases. These findings corroborate previous studies demonstrating that nondisjunction events, particularly during maternal meiosis, represent the primary mechanism responsible for the extra chromosome [17-19].

A noticeable male predominance was observed in our cohort, with males comprising 60.6% of DS cases. Similar sex-based differences have been reported in epidemiological and cytogenetic studies from diverse populations [7,20]. However, other studies have described approximately equal sex ratios or slight female predominance in certain cohorts [21]. Comprehensive clinical descriptions of DS and its multisystem involvement have been extensively documented in review literature [22]. The observed male predominance has been hypothesized to reflect potential biological mechanisms, including differences in meiotic nondisjunction patterns or intrauterine survival [17].

Structural chromosomal abnormalities, particularly Robertsonian translocations, accounted for a substantial subset of trisomy 21 cases. While rob(14;21) remains the most common translocation subtype, an important observation in this cohort was the near-comparable frequency of rob(21;21). This finding contrasts with most published series, which report rob(14;21) comprising approximately 80%-85% of Robertsonian translocations [2,7]. The relatively higher proportion of rob(21;21) observed here may reflect population-specific cytogenetic characteristics or referral bias in a large diagnostic setting. This observation is clinically significant, as inherited rob(21;21) translocations are associated with an almost 100% recurrence risk of trisomy 21 [23], underscoring the importance of routine parental karyotyping and comprehensive genetic counseling. Identification of rob(21;21) is particularly noteworthy given its rarity, with only limited reports available in the literature, including isolated case descriptions [23].

Uncommon chromosomal abnormalities such as deletions at 5p15.3, duplications, satellite polymorphisms (pstk+ and ps+), and heterochromatic variations (qh+ and qh−) were observed, highlighting the broad cytogenetic heterogeneity associated with DS. These findings are generally considered cytogenetic polymorphisms rather than primary etiological factors; however, they may complicate karyotypic interpretation in diagnostic settings. Satellite associations and heterochromatic variants have previously been reported in DS and may influence chromosomal behavior during cell division [24].

Mosaic trisomy 21 was identified in a small proportion of cases, consistent with previous reports indicating that approximately 2%-4% of individuals with DS exhibit mosaic forms of the condition [25,26]. Germ-line transmission of trisomy 21 has also been described, suggesting potential contributions of trisomy rescue mechanisms and grandmaternal age effects in selected families [27].

Overall, these findings reinforce that DS represents a genetically heterogeneous condition with diverse cytogenetic mechanisms. Male predominance, varied Robertsonian translocations, chromosomal inversions, heterochromatic polymorphisms, and mosaicism together create a complex cytogenetic landscape. Advanced molecular cytogenetic and genomic approaches may further elucidate nondisjunction mechanisms, recurrence risks, and genotype-phenotype correlations in future studies.

Limitations of the study

This study has certain limitations that should be considered when interpreting the findings. First, the retrospective nature of the analysis limited the availability and uniformity of detailed clinical information, including maternal age at conception, parental karyotypes, pregnancy outcomes, and long-term phenotypic follow-up. Consequently, causal associations between specific cytogenetic findings and clinical severity could not be assessed.

Second, the cohort represents individuals referred to a tertiary diagnostic laboratory, which may introduce referral bias and limit direct extrapolation of frequencies to the general population. However, the large sample size, extended study duration, and inclusion of cases from a broad geographic region likely mitigate this effect and provide a representative overview of the cytogenetic spectrum encountered in routine clinical practice.

Third, cytogenetic analysis was performed using conventional GTG-banded karyotyping, which remains the diagnostic gold standard for detecting numerical chromosomal abnormalities and balanced structural rearrangements in DS. Nonetheless, this approach has limited resolution for identifying submicroscopic copy-number changes or gene-level variants. Advanced molecular techniques such as chromosomal microarray or genome-wide sequencing were not routinely applied and could provide additional insights into genotype-phenotype correlations in future studies.

Finally, parental karyotyping was not available for all cases with Robertsonian translocations, limiting the ability to distinguish de novo from inherited rearrangements and to directly estimate recurrence risk. Despite this, the identification and characterization of translocation subtypes, particularly the relatively higher frequency of rob(21;21), remain clinically relevant and emphasize the importance of detailed cytogenetic evaluation for genetic counselling.

Overall, while these limitations exist, they do not detract from the principal findings of the study. The large cohort size, standardized laboratory protocols, high metaphase resolution, and ISCN-compliant reporting provide robust insights into the cytogenetic heterogeneity of trisomy 21 in a large Indian referral population.

## Conclusions

DS remains the most common autosomal aneuploidy, primarily caused by meiotic nondisjunction. Male predominance and increased prenatal referrals highlight the growing importance of cytogenetic analysis in genetic diagnosis. Integrating cytogenetic evaluation with clinical and molecular data is essential for accurate diagnosis, counselling, and informed reproductive decision-making. A higher frequency of rob(21;21) translocations was observed, underscoring the need for detailed cytogenetic evaluation, routine parental karyotyping, and targeted genetic counseling to enable accurate recurrence risk assessment in affected families.
